# Effectiveness of online mindfulness interventions on medical students’ mental health: a systematic review

**DOI:** 10.1186/s12889-021-12341-z

**Published:** 2021-12-18

**Authors:** Vaidehi Yogeswaran, Christo El Morr

**Affiliations:** School of Health Policy and Management, York University, Toronto, Ontario Canada

**Keywords:** Mindfulness, Mental Health, Burnout, Depression, Anxiety, Stress, Medical Students, Residents

## Abstract

**Background:**

Medical school typically presents students with a combination of academic and personal stressors that may lead to substandard mental health wellbeing. Meditation practices such as mindfulness facilitate a greater awareness of one's thoughts and feelings, thereby decreasing emotional reactivity. The use of mindfulness-based interventions delivered online has considerable potential in fostering self-care and helping medical students to handle mental health challenges. We examined the available evidence on the use of online mindfulness interventions in order to determine whether they are feasible and effective for improving medical students’ mental health.

**Methods:**

We performed a systematic review guided by PRISMA guidelines and utilised the following databases: ProQuest, Medline, PubMed, PsycINFO, Web of Science, IEEE Explore, Cochrane, and CINAHL. The key search terms used include mindfulness, cognitive behavioural therapy, acceptance and commitment therapy, online, web, virtual, internet cyber, app, medical students, residency students, and residents. English-language articles published in the last ten years that described online interventions for medical students or residents were included in the review.

**Results:**

Two studies describing the impact of online mindfulness interventions on medical students’ mental health were identified. Research in this domain is nascent; available qualitative and quantitative evidence suggests benefits in self-compassion, perceived stress, cognitive skill use, mindfulness, creating coping mechanisms, and greater awareness of emotions and feelings. There was no evidence of the effectiveness of online mindfulness interventions on depression, anxiety and burnout. There was, however, general low program usage and participation tended to diminish near the conclusion of the interventions.

**Conclusions:**

The evidence found in the systematic review exhibits the potential for online mindfulness interventions to be effective in addressing some mental health challenges of medical students. There was insufficient evidence to support the use of online mindfulness interventions for burnout, depression, and anxiety. Longitudinal studies with randomised controlled trials are required to generate stronger and robust evidence.

**Supplementary Information:**

The online version contains supplementary material available at 10.1186/s12889-021-12341-z.

## Introduction

The journey to medical school graduation presents students with high levels of stress, burnout, and poor mental health and wellbeing. A growing body of research suggests that medical students are at high risk of experiencing numerous psychological illnesses. The heightened levels of stress that accompany medical training and the subsequent junior doctor years can lead to anxiety, burnout, depression, suicidal thoughts, and alcohol abuse [1]. Medical students notice signs of burnout and psychological distress even before graduation and suffer from more psychological distress than those with a similar age profile [2]. The combination of academic and personal stressors can give rise to elevated levels of depressive symptoms, which can culminate in poor mental wellbeing [3]. In fact, compared to the general population, depression and depressive symptoms are more common among medical students, with a prevalence rate estimated at 27.2 vs. 9.3%-7.2% in the general population [4]. Medical school presents an increase in psychological distress and a decline in both life satisfaction and self-reported empathy [2]. The multiple stressors faced by medical students coupled with substandard mental health and high rates of burnout may ultimately harm their ability to bond with patients and provide empathetic care [3].

The strenuous lifestyle led by medical students bolsters the idea of introducing practices that assist with emotion regulation and that encourage self-care. Mindfulness has been described as a process of bringing attention to experience life moment-by-moment [5]. While mindfulness can modify one’s attitude towards thoughts leading to less influence of thoughts on one’s subsequent feelings and behaviors, cognitive behaviour therapy (CBT) aims at restructuring cognition to develop more balanced and functional ways of approaching the environment [6, 7]. Mindfulness meditation uses various techniques to attain a state of mindfulness and was proven to be a means to reduce emotional reactivity by guiding one's attention to their thoughts and feelings [8]. It has slowly become well known over the past years as a strategy to enhance emotional wellbeing and handle stress. Continuous practice of mindfulness may promote higher levels of self-compassion, stress regulation, and effective coping [1]. Systematic reviews conducted on the effectiveness of meditation programs and mindfulness-based intervention programs led to improvements in resilience to stress, anxiety and depression among university students and other populations, whether delivered offline [9] or online [10–14]. Moreover, practicing mindfulness may also inspire empathy and compassion for other individuals [1].

Cognitive behavioural therapy (CBT) is another emerging strategy that has proven to be effective in the treatment of a range of mental health issues [15]. Cognitive behavioural stress management programs are shown to be effective in increasing perceived stress management competency, self-efficacy, and self-esteem [16, 17]. Additionally, web-based acceptance and commitment therapy (ACT), a form of cognitive behavioural therapy, was found to be successful in alleviating the academic worries and wellbeing of a group of college students [18]. A recent systematic review presented evidence of the effectiveness of online mindfulness interventions on mental health [10], and a recent randomised control trial among university students found mindfulness-based cognitive behavioural therapy to be effective in reducing depression, anxiety and stress while increasing wellbeing [11–14, 19, 20].

Therapy centres and mental health program initiatives tend to be confined to larger metropolitan areas. Mental health programs delivered online can substantially increase accessibility to mental health interventions remotely for a wide geographic area, overcoming limitations of distance and time, and online mindfulness offers an opportunity to circumvent those limitations [21]. E-Health has been effective in providing health services in distributed and rural areas [22], and online interventions can be especially beneficial for students learning in distributed or rural facilities [1]. Moreover, compared to traditional interventions, online programs provide more privacy and flexibility [20]; since the programs are online, students have the option to access them at any time from the privacy of their rooms if they wish to [23]. It is also important to note that most medical students are accustomed to using technology and regularly consult the internet to obtain information relating to their health [15]; the ease with which they use technology makes them ideal consumers who would be able to derive the full benefit from online mindfulness interventions [15]. In addition, online interventions allow the limited number of individuals qualified in mindfulness coaching to be overcome [1].

Given the increase in mental health disorders experienced by medical students and the effectiveness of online mindfulness programs in general, and among university students in particular, the impact of these interventions on medical students warrants deliberation. Although there is a fair amount of research relating to mindfulness programs for students [10], there remains a paucity of literature concerning online interventions for medical students. Thus, it is important to synthesise the available evidence regarding the use of online mindfulness interventions in improving the mental health of medical students. To our knowledge, there is currently no systematic review summarising the current evidence. To mitigate this knowledge gap, we conducted a systematic review of literature on online mindfulness interventions for medical students. The study objective was to investigate whether online mindfulness interventions can be utilised to promote mental health for medical students. The results of the review will be used to advise future online mindfulness interventions targeting medical students.

## Methods

### Search strategy

A systematic review was conducted in the following eight online databases: ProQuest, Medline, PubMed, PsycINFO, Web of Science, IEEE Explore, Cochrane, and CINAHL. The literature was assessed by employing a comprehensive search strategy based on systematically screening titles and using key search terms identified with the support of a librarian. The following search terms were included: mindfulness, cognitive behavioural therapy, acceptance, and commitment therapy, online, web, virtual, internet cyber, app, medical students, residency students, and residents. Six citations were extracted, three of which were not duplicates. The full search strategies can be found in the Appendix.

### Eligibility criteria

The inclusion criteria were predetermined and focused on studies that were published in the last ten years, described online mindfulness interventions, and if the study population was limited to medical students or residents. The exclusion criteria included studies that described interventions that were not delivered online, targeted students that were not in medical school, were not written in English, did not have full texts, or were excerpts from books. The most recent search took place on 28 October 2020. The protocol of this review was not registered. The 10-year window for the search was based on the fact that a previous comprehensive systematic review about online mindfulness interventions identified the first online mindfulness intervention in 201 3[10].

### Selection process

The inclusion and exclusion criteria were first applied to 3 abstracts and the 3 were identified as potentially eligible. The full-text versions were retrieved for the 3 articles and reviewed by both authors for the final selection. The 3 articles were selected for systematic review and synthesis. References of these articles were also searched, but no additional articles were identified. While our main interest in terms of outcome was depression, anxiety stress and burnout, we did not base our selection on those outcomes only, and outcome was not part of the inclusion/exclusion criteria. The literature search, review, and data collection from articles was conducted by a single individual and was repeated by one other individual. The resulting articles from the two searches were then integrated. Ambiguities and disagreement were resolved through discussion and consensus. We have planned to assess the risk of biases by investigating confounding, selection bias, exposure assessment, outcome measurement, and missing data. A meta-analysis was not conducted because of the paucity of articles and the disparities in study design, variables, and exposures between the studies.

## Results

**Fig. 1:**
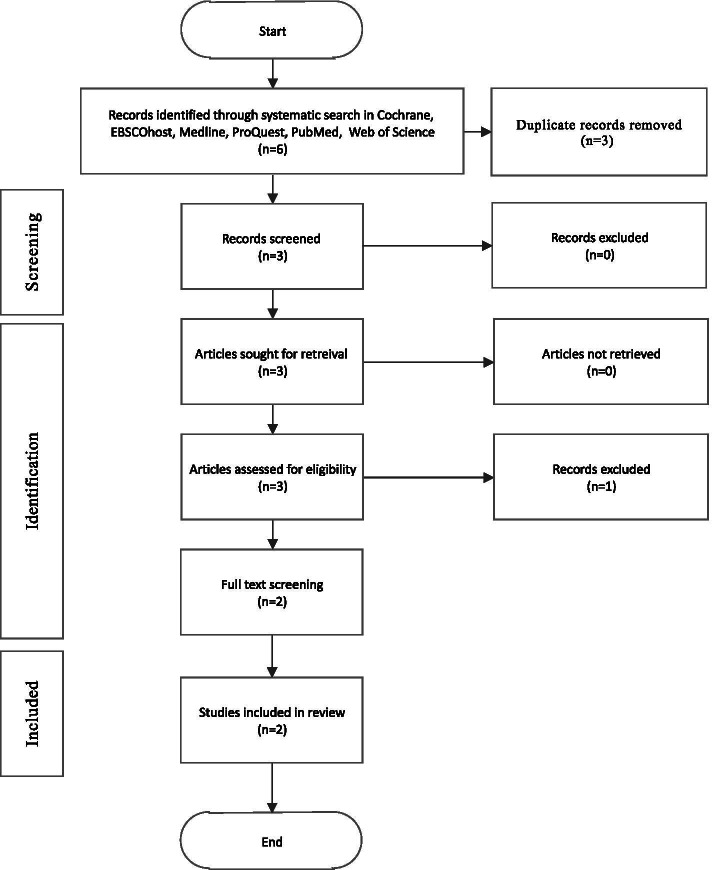
Flow chart for article identification and selection

In total, six articles were identified, three of which were duplicates. Of the three unique articles, none were excluded based on the content of their abstracts. The inclusion criteria were applied to the three articles after their full text was read, and two articles were kept for analysis [1, 23] (Fig. 1). Table 1 lays out the studies in terms of population, intervention, comparison groups, and outcomes (PICO).

**Table 1:** Population, intervention, comparison groups, and outcomes (PICO) table

Author/Year	Population	Intervention (Study design, time horizon, perspective)	Control group	Outcome (Results)	Items of interest
Sarah Moore, Rita Barbou, Hanh Ngo, Craig Sinclair, Richard Chambers, Kirsten Auret, Craig Hassed & Denese Playford (2020)	Medical students at a Rural Clinical School *N*=47	**Intervention Type:** Single-arm prospective mixed method cohort study **Intervention:** MTP (Online mindfulness training programme) Short mini-lectures Guided meditations **Duration:** 8 weeks **Follow Up:** 4 months **Measurement tools:** Perceived Stress Scale (PSS) Self Compassion Scale (SCS) Compassion Scale (CS) Completed at baseline, completion of intervention, 4 months follow up	No control group	Many participants reported mindfulness: ● Increased awareness of nature of mind ● Provided opportunities to acknowledge thoughts and emotions in a judgmental manner allowing participants to respond in more controlled way ● Helpful in developing self-compassion and compassion for others ● Allowed participants to become more present, thereby reducing stress ● Some reported practising mindfulness helped to improve productivity and performance No immediate post-training impact on stress levels Impact seen more clearly at 4 month follow up, just before final exams	Medical students experience stress during training As stress continues into junior doctor years, this may lead to burnout, anxiety, depression, suicidal thinking, alcohol abuse
Danilewitz M., Koszycki D., Maclean H., Sanchez-Campos M., Gonsalves C., Archibald D., and Bradwejn J. (2018)	Medical students *N*= 52	**Intervention Type:** Prospective pilot cohort design **Intervention:** MIND-MED Comprised of 7 online modules that explain the foundations of mindfulness practice and address themes linked to medical student experiences Each module was sequential and contained video content and meditation practice Each module lasted 25-35 minutes **Duration:** 7 weeks to 4 months Based on participant rate of progression **Measurement Tools:** Jefferson Empathy Scale-Medical Students version Self-Compassion Scale-Short Form Five Facet Mindfulness Questionnaire	No control group	**Feasibility** was easily achieved Module completion was high ● 86.5% finished at least 1 module ● 22.2% finished 1-3 modules ● 16.7% finished 4-6 modules ● 66.7% finished all 7 modules Regular daily practice of meditation techniques was low **Estimated Marginal Mean for Pre- and Post-Intervention Self-Report Measures (95% Confidence Intervals** ● Improvement from baseline for burnout was not statistically significant ● Increase in empathy levels but not significant ● Absence of statistically significant changes in empathy may be rooted to JES-S scale which mainly measures attitude towards empathy ● Statistically significant increase for self-compassion ○ Pre-intervention: 35.0+-1.2 ○ Post-intervention: 39.3+-1.3 ● Statistically significant increase for “observe” and “describe” facets of mindfulness ● Observe: ○ Pre-intervention: 12.9+-0.5 ○ Post-intervention: 15.1+-0.5 ● Describe: ○ Pre-intervention: 16.7+-0.5 ○ Post-intervention: 18.4+-0.6 The module that the highest percentage of participants (45.1%) found to be the most relevant Most number of participants: Module 5 (Self Acceptance: Dealing with Perfectionism Relating to Our Judging Mind) Most number of participants (53.6%) found support materials for Module 7 (Moving Beyond the Program) to be the most useful	No significant effect on burnout levels

## Studies’ characteristics

### Age

The mean age of participants in the study by Moore et al. was 26.7, which is higher than that of the other two studies [1]. The standard deviation was 3.9.The ages of participants in the study by Danilewitz et al. ranged from 20 to 37 years, with the mean age being 23.8 years ± 2.7 [23].

### Gender distribution

Of the 47 students that participated in the study by Moore et al., nine (19.15%) were male and 38 (80.85%) were female [1]. Thirty-six of the 52 students (69.2%) who decided to participate in the Danilewitz et al. study were female [23].

### Settings

The intervention in Moore et al.’s study was conducted on students from an Australian Rural Clinical School who lived in one of fourteen rural towns for the duration of the school year [1], while ﻿Danilewitz et al.’s study took place at the University of Ottawa, in the capital city of Canada [23].

### Population

The study by Moore et al. was conducted on students in their penultimate academic year. Conversely, the study performed by Danilewitz et al. included students from a range of year levels [23]. Of the 52 participants in the study by Danilewitz et al., 21 (40.39%) were in their first year and 20 were in their second year (38.46%) [23]. Ten students (19.23%) were in their third year while one was in their fourth year (1.92%) [23].

### Ethnicity

The largest ethnic group found in Moore et al. was Caucasian or white [1, 15]. Along with 36 Caucasian students (76.60%), four Asian students (8.50%), one Latin American student (2.13%), and six students (12.77%) of unnamed ethnicity (i.e., “other (incl. Indian)”) were enrolled in Moore et al.’s study [1]. Ethnicity of participants was not reported in the study by Danilewitz et al. [23].

## Studies’ designs and outcomes

### Intervention design and length

The length and type of intervention varied between the two studies. Moore et al.’s study took place over eight weeks and was a single-arm prospective mixed method cohort study [1]. The mindfulness intervention utilised in Danilewitz et al.’s pilot study was entirely dependent on the time taken by the participant to complete the seven modules [23]. Overall, the intervention duration time varied from seven weeks to four months [23].

### Mindfulness components

The Mindfulness Training Program (MTP) employed in the study by Moore et al. was comprised of two components: a mini lecture and a guided mindfulness meditation session. The mini lectures took place for ten minutes every week and explained various mindfulness concepts such as mindfulness communication, decreasing distractions and procrastination, emotion regulation, and compassion. The five-minute guided mindfulness meditation session occurred daily and involved activities that ranged from mindful breathing to mountain meditation [1].

The MIND-MED intervention employed in the Danilewitz et al. study included seven online modules that covered the basics of mindfulness and topics relating to medical student experiences. Each module was approximately 25-30 minutes long and contained video content and meditation practice. Downloadable audio recordings of various meditation practices and pertinent reading material were posted on the website. Participants were requested to note the amount of time they allocated each week to partaking in mindfulness activities [23].

### Intervention effects on mental health outcomes

The two studies used various outcome measurement tools; Table 2 summarises the outcome measurement tools used in each of the studies.

**Table 2:** Measurements tools used

Study	Mindfulness	Stress	Self Compassion	Empathy	Burnout
Moore et al. (2020)		PSS	SCS	CS	
Danilewitz et al. (2018)	FFMQ		JSE (S- version)	SCS-SF	MBI

*CS* Compassion Scale*, FFMQ* Five Facets Mindfulness Questionnaire*, JSE (S-version)* Jefferson Scale of Empathy Medical Students version*, MBI* Maslach Burnout Inventory*, PSS* Perceived Stress Scale*, SCS* Self Compassion Scale*, SCS-SF* Self-Compassion Scale – Short Form (SCS-SF)

Quantitative evaluation of the intervention in Moore et al. demonstrated statistically significant changes in PSS and SCS scores from baseline to the four-month follow-up assessment. The PSS scores of participants had significantly decreased by two points at the four-month follow-up. Additionally, participants’ SCS scores had significantly increased at the eight-week mark as well as at the four-month follow-up (*p*=0.05). SCS scores had increased by 6.3 points by the end of the eight-week program and 5.5 points at the four-month follow-up (*p*=0.05). There were no significant changes in CS scores [1].

A strong baseline effect was also noted for changes in PSS and SCS scores. Participants who experienced more stress at baseline had a more substantial decrease in stress at the four-month follow-up (*p*=0.0005). Those who reported lower self-compassion at baseline experienced an increase in self-compassion after four months (0.0062) [1].

Qualitative evaluation of the intervention revealed that several participants derived benefits from the program in two main areas: engagement with the MTP and the impact of mindfulness on the personal and professional lifestyles of participants. In terms of engagement, a few participants reported that they were more encouraged to create coping mechanisms for anxiety and stress management. They were also motivated to learn about strategies that would enhance their performance at school. Although it was difficult to maintain engagement with the program with a heavy academic workload, participants were able to recognise the benefits of mindfulness [1].

Impact-wise, participants noted that mindfulness helped to facilitate a greater awareness of the nature of the mind. It offered opportunities for participants to recognise emotions and thoughts in a non-judgemental manner, which accordingly allowed for more regulated responses [1]. The participants were also able to develop more self-compassion and compassion for other individuals. Moreover, practising mindfulness led to improvements in productivity and performance and participants were able to become more present, thereby reducing stress. While there was not a significant impact on stress levels after the program, the four-month follow-up that occurred before final exams revealed that stress levels had reduced. The program also resulted in elevated self-compassion levels at program completion and at the four-month follow-up as well. In terms of feasibility, participants only practiced mindfulness once a week by week 8 of the program. Nevertheless, it is feasible to implement a short MTP intervention delivered online as it still encouraged students to practice mindfulness weekly despite heavy academic work [1].

Results from the Danilewitz et al. study [23] demonstrated no statistically significant changes in the MBI’s burnout subscales, including Personal Achievement (*p*=0.55), Emotional Exhaustion (*p*=0.51) and Depersonalisation (0.71). There was also no significant change in JSE-S scores (*p*=0.06). The intervention, however, had a statistically significant impact on the “describe and observe” facets of the Five Facet Mindfulness Questionnaire. The describe facet refers to the ability to describe feelings and emotions. The estimated marginal mean for this facet increased from 16.7 ± 0.5 to 18.4 ± 0.6 (*p*<0.001). The observe facet describes the ability to notice the physical feelings that arise from certain activities, such as the sensations that one feels from moving their body during a walk [23]. The estimated marginal mean for this facet increased from 12.0 ± 0.5 to 15.1 ± 0.5 (*p*<0.001). Additionally, the estimated marginal mean ratings for self-compassion had a statistically significant increase from 35.0 ±1.2 to 39.3 ±1.3 (*p*=0.001).

### Program use

Program use was not reported in Moore et al. [1]. In the Danilewitz et al. study, at least half the participants practiced mindfulness three days or more per week in the first three weeks of the intervention, and then their practice dropped. About half of the students were practicing for around 30 minutes per week by week 8. At the four-month follow-up, 32% of the participants practiced at least once weekly; however, 89% practiced for 10 minutes or less per session [23].

Table 3 provides a summary of the above results.

**Table 3:** Summary of results in the two studies

Study	Moore et al. (2020)	Danilewitz et al. (2018)
Sample	47	52
Age mean (SD) [Range]	26.7 (3.9) Range not reported	23.8 years (2.7) [20 to 37]
Gender Female vs. Male	80.85% vs. 19.15%	69.2% vs. 30.8%
Program Use (Female vs Male)	No reported	No reported
Settings	Rural	Urban
*Population*	Students in their penultimate year	1st to 4^th^ Year medical students
*Ethnicity* *Caucasian vs other*	76.60% vs 23.40%	Not reported
*Intervention Design*	Single arm Prospective pilot cohort design	Single arm Prospective pilot cohort design
*Intervention Length*	8 weeks	7 to 12 months (based on participant rate of progression through the modules)
*Mindfulness Components*	Weekly: one 10-minutes mini lecture and Daily: 5-minutes guided mindfulness meditation sessions	Downloadable audio recordings of different meditation practices of varying durations (e.g., 15- or 30-minute body scan meditation) For mindful audio and video yoga postures Reading material about mindfulness and student wellbeing Weekly email reminders
Effects on Mental Health Outcomes	Follow-up: 21 Significant change PSS and SCS scores No Significant change CS	Follow-up N=45 Significant change JSE-S (s-version) FFMQ: Describe and Observe facets SCS-SF No Significant change MBI (including all 3 dimensions)
Actual Program Use	Amount of practice were not significantly correlated with changes in scores on PSS, SCS, and CS First 3 weeks • 50% practiced mindfulness 3 days or more per week Last 5 weeks • About 50% practiced around 30 minutes per week by Week 8. At 4-month • 32% continued to practice at least once weekly • 89% of which practiced for 10 min or less per session	45 (86.5%) completed at least one module. • 10 out of 45 (22.2%) completed one to three modules, • 7 out of 45 (16.7%) completed four to six modules, • 28 out of 45 (66.7%) completed all seven modules

## Discussion

This systematic review of studies on mindfulness-based online interventions targeting medical students showed that there are very few studies in their first stages and address different facets of mental health. Overall positive impact of the describe and observe facets of the FFMQ, and on empathy, was noted in one study [23]. The program use was overall medium to high. None of the study designs is a randomized control trial, and all ran with no control group, hence the results are weak and subject to bias since with absence of randomization random sequence generation, allocation concealment, blinding of participants and personnel, and blinding of outcome assessment were not possible, furthermore, data was incomplete as the rate of the student who did not complete the studies were 55.32% in the Moore et al. study [1] , and 13.46% Danilewitz et al. [23].

### Quality of the studies

The two studies are designed as one arm and as pilot studies. A randomised control trial is a much-needed intervention for medical students to establish strong evidence of the possible effectiveness of online mindfulness for this population. Randomized control trials using online mindfulness interventions addressing students’ mental health have proven to be effective [11, 12, 14, 24–26]; hence, implementing RCTs targeting medical students seems a logical next step to build high quality strong evidence supporting such approaches for medical students.

### Interventions’ use

Participants in the MTP intervention had favourable attitudes and opinions regarding the program [1]. Although program engagement dropped near its termination, several students acknowledged its value and benefits [1]. The students in the MTP program might have been able to fully realise its benefits and they would have been more invested in an online mindfulness program due to the lack of mental health services in rural areas.

Although it is feasible to conduct online mindfulness interventions, the general low program usage across the two interventions necessitates deliberation regarding program content. A mindfulness program at Monash University, a public university in Australia, achieved high adherence rates [1] despite being delivered mostly online. The inclusion of face-to-face time with session facilitators and the longer program time were driving factors in increasing participant engagement [1]. It might be helpful in future studies to include virtual therapy or counselling sessions in online mindfulness programs as they allow participants to interact and communicate with mental health professionals without having to seek out these services in person. Students would be able to build a close relationship with professionals, which may ultimately contribute to high program adherence rates. For example, despite high module completion rates for the MIND-MED program, consistent daily practice of meditation mindfulness techniques remained low. Cultivating a strong bond with mental health professionals might help encourage students to maintain their mindfulness practice.

### Online mindfulness

While online mindfulness interventions have shown an effect on mental health, little research has been done on the effect of online mindfulness on medical students. The particular challenges medical students face and the fast-paced environment they work in substantiate the need to test online mindfulness interventions dedicated to this group of students. Since a video-based intervention was found to be effective in one study addressing university students [14], building video-only-based interventions seems an interesting approach that has the potential to substantially decrease the cost of the intervention and large-scale solution deployment.

## The community dimension in online interventions

Currently, the community dimension of online presence has not been addressed in online mindfulness interventions. Online communities [27, 28], whether static or mobile, proved to play a major role in digital health [29–31], including mental health [11, 32, 33]. They have been proven to be effective in knowledge dissemination [34], health monitoring [35], self-management of health conditions [36] and chronic disease management [37].

Besides, adherence in online studies proved to be challenging, the virtual community dimension brings the presence of peers and their social support, which was proven to help in commitment to intervention and to positively influence outcomes.

While in one study online forums were incorporated but were not effectively used by online community members [12, 38, 39], the sense of online community and its effect on mental health outcomes have not been addressed in any study. Moreover, Covid-19 has had a drastic impact on the population’s mental health [40–43], including medical students [44–48]. Specifically, isolation had a major impact on the mental health of the population in general [49] and medical students in particular [50], so studying the effect of the sense of belonging to a community and the sense of isolation on mental health outcomes appears to be paramount. There are advanced techniques that might become useful in online communities, such as online agents [51, 52] that allow notification based on user preferences [27, 35, 53–56]. It is important to note that frameworks to evaluate the successful implementation and adoption of online communities do exist [57].

### User experience

One of the missing aspects in all studies is the formal evaluation of the user interface and the user experience. Such evaluations are usually overlooked because of the time and cost they require. However, the successful adoption of a solution deployed in the real world depends on the user interface ease of use and the user experience; until a formal evaluation is performed there is little evidence that an intervention can pass from the prototype phase to a full deployment phase. Currently, the user experience evaluation is less onerous and is conducted quickly and robustly using design iterations that proved to be just as informative as the traditional, more time-consuming evaluation paradigms, including in the context of virtual communities [53], using fast-paced methodology such as lean UX [58].

### Analytics

Finally, healthcare analytics that allow the collection of objective data [59–61], such as app or website use, to predict healthcare outcomes using machine learning approaches [60, 62] constitute an approach that has been overlooked so far in online mental health interventions. Analytics can drive intelligent indicators [63] to build smart mindfulness virtual communities [64] that display indicators and predictors using user-friendly visualisation tools [65]. The presence of analytics and machine learning would be important to explore in future online mindfulness interventions targeting medical students.

### Strengths and limitations

This is the first systematic review to address the effectiveness of online mindfulness interventions for medical students’ mental health; it revealed a paucity of research in the domain and the need for interventions targeting medical students given the particular mental health challenges they face. Research in this domain is nascent, and more research is needed to draw robust conclusions. However, this study has some limitations. One of these limitations is that the search was limited to the English language; there could be studies in other languages that were missed. Another limitation was the difficulty of comparing the results, given the paucity of research in the field and the variation in the outcome measurement tools. Given this variation, our review shows that there are limits for comparing the effectiveness of the different interventions on medical students’ mental health.

## Conclusions

The evidence reviewed suggests that online mindfulness interventions targeting medical students have the potential to be effective in reducing symptoms of stress and increasing self-compassion, empathy, and mindfulness. There is no evidence to support an effect on depression or anxiety. There is a lack of strong evidence about the effectiveness of such interventions; further studies, and more specifically randomised control trials, are needed to establish such effectiveness. In this regard, web-based as well as mobile-based (i.e. m-Health) interventions could be explored [32, 57], as could virtual communities’ role in providing a sense of community.

Online interventions seem to be an attractive option due to the wide use of smartphones among medical students. Research addressing the usability and safety of online mindfulness communities is virtually non-existent; there is a need to find answers to the unintended consequences of the use of ICT [58]. Future longitudinal follow-ups for a longer time (e.g., one year) could help determine the long-term effects of the use of online mindfulness interventions.

## Supplementary Information

**Additional file 1.**

## Data Availability

Data sharing is not applicable to this article as no datasets were generated or analysed during the current study.

## Abbreviations

UX: User Experience
CBT: Cognitive behavioral therapy
ACT: Acceptance and commitment therapy
PICO: Population, intervention, comparison groups, and outcomes
