# Supplementary material for: Effectiveness of online mindfulness interventions on medical students’ mental health: a systematic review
Source: BMC Public Health. 2021 Dec 18;21:2293. doi: 10.1186/s12889-021-12341-z (PMC8683314; doi:10.1186/s12889-021-12341-z)
Supplement: Supplementary file 1 — Additional file 1. [file 12889_2021_12341_MOESM1_ESM.docx]

# Appendix 1: search strategies

## 1. ProQuest

S4 ((ti(Medical students) OR ti(residents) OR ti(residency students)) AND PEER(yes)) AND ((ti(cyber) OR ti(web) OR ti(virtual) OR ti(online) OR ti(app) OR ti(internet)) AND PEER(yes)) AND ((ti(mindfulness) OR ti(cognitive behavioral therapy) OR ti(acceptance AND commitment therapy)) AND PEER(yes))

S3 ti(mindfulness) OR ti(cognitive behavioral therapy) OR ti(acceptance AND commitment therapy)Limits applied

S2 ti(cyber) OR ti(web) OR ti(virtual) OR ti(online) OR ti(app) OR ti(internet)Limits applied

S1 ti(Medical students) OR ti(residents) OR ti(residency students)Limits applied

## 2. Medline

1 Students, Medical/ or Education, Medical, Undergraduate/ or Education, Medical, Graduate
2 limit 1 to (english language and yr="2010 - 2020")

3 Cyber/ or Internet/ or Web Browser/ or virtual/ or online/ or app/ or internet/

4 (acceptance and commitment therapy).mp. [mp=title, abstract, original title, name of substance word, subject heading word, floating sub-heading word, keyword heading word, organism supplementary concept word, protocol supplementary concept word, rare disease supplementary concept word, unique identifier, synonyms]
5 1 and 2 and 3 and 4

## 4. PsychINFO

((ti(Medical students) OR ti( residents) OR ti(residency students )) AND PEER(yes)) AND ((ti(cyber) OR ti(web) OR ti(virtual) OR ti(online) OR ti(app) OR ti(internet)) AND PEER(yes)) AND ((ti(mindfulness) OR ti(cognitive behavioral therapy) OR ti(acceptance AND commitment therapy)) AND PEER(yes))

## 5. Web of Science

# 4 #3AND #2 AND #1
# 3 (TI=(mindfulness OR cognitive behavioral therapy OR acceptance "and" commitment therapy) ) AND LANGUAGE: (English)

# 2 (TI=(cyber OR web OR virtual OR online OR app OR internet) ) AND LANGUAGE: (English)

# 1 (TI=(Medical students OR resident* OR residency student*) ) AND LANGUAGE: (English)

## 6. CINAHL

S4 S1 AND S2 AND S3
S3 TI medical students OR TI residents OR TI residency students
S2 TI cyber OR TI web OR TI virtual OR TI online OR TI app OR TI internet
S1 TI mindfulness OR TI cognitive behavioral therapy OR TI (acceptance and commitment therapy)

## 7. IEEE Xplore

4 [((((((Mesh_Terms:mindfulness) OR Mesh_Terms:cognitive behavioral therapy) OR Mesh_Terms:acceptance and commitment therapy) refined by:Year:2010-2020 )) AND (((((((Mesh_Terms:web) OR Mesh_Terms:cyber) OR Mesh_Terms:online) OR Mesh_Terms:web) OR Mesh_Terms:app) OR Mesh_Terms:internet) refined by:Year:2010-2020 )) AND ((((Mesh_Terms:medical students) OR Mesh_Terms:residents) OR Mesh_Terms:residency students) refined by:Year:2010-2020 )](https://ieeexplore-ieee-org.ezproxy.library.yorku.ca/search/searchresult.jsp?contentType=all&sortType=&searchField=Search_All&combineQuery=ranges%3D2010_2020_PublicationYear%26matchBoolean%3Dtrue%26searchField%3DSearch_All%26queryText%3D%28%28%28meshTerms%3Amindfulness%29+OR+meshTerms%3Acognitive+behavioral+therapy%29+OR+meshTerms%3Aacceptance+and+commitment+therapy%29.OPAND.ranges%3D2010_2020_PublicationYear%26matchBoolean%3Dtrue%26searchField%3DSearch_All%26queryText%3D%28%28%28%28%28%28meshTerms%3Aweb%29+OR+meshTerms%3Acyber%29+OR+meshTerms%3Aonline%29+OR+meshTerms%3Aweb%29+OR+meshTerms%3Aapp%29+OR+meshTerms%3Ainternet%29.OPAND.ranges%3D2010_2020_PublicationYear%26matchBoolean%3Dtrue%26searchField%3DSearch_All%26queryText%3D%28%28%28meshTerms%3Amedical+students%29+OR+meshTerms%3Aresidents%29+OR+meshTerms%3Aresidency+students%29&history=no) You Refined By: Year: 2010-2020

3 [((("Mesh_Terms":medical students) OR "Mesh_Terms":residents) OR "Mesh_Terms":residency students)](https://ieeexplore-ieee-org.ezproxy.library.yorku.ca/search/searchresult.jsp?contentType=all&ranges=2010_2020_PublicationYear&matchBoolean=true&searchField=Search_All&queryText=(((meshTerms:medical+students)+OR+meshTerms:residents)+OR+meshTerms:residency+students)&history=no) You Refined By: Year: 2010-2020

2 [(((((("Mesh_Terms":web) OR "Mesh_Terms":cyber) OR "Mesh_Terms":online) OR "Mesh_Terms":web) OR "Mesh_Terms":app) OR "Mesh_Terms":internet)](https://ieeexplore-ieee-org.ezproxy.library.yorku.ca/search/searchresult.jsp?contentType=all&ranges=2010_2020_PublicationYear&matchBoolean=true&searchField=Search_All&queryText=((((((meshTerms:web)+OR+meshTerms:cyber)+OR+meshTerms:online)+OR+meshTerms:web)+OR+meshTerms:app)+OR+meshTerms:internet)&history=no) You Refined By: Year: 2010-2020

1 [((("Mesh_Terms":mindfulness) OR "Mesh_Terms":cognitive behavioral therapy) OR "Mesh_Terms":acceptance and commitment therapy)](https://ieeexplore-ieee-org.ezproxy.library.yorku.ca/search/searchresult.jsp?contentType=all&ranges=2010_2020_PublicationYear&matchBoolean=true&searchField=Search_All&queryText=(((meshTerms:mindfulness)+OR+meshTerms:cognitive+behavioral+therapy)+OR+meshTerms:acceptance+and+commitment+therapy)&history=no) You Refined By: Year: 2010-2020

## 8. COCHRANE

1 (mindfulness or cognitive behavioral therapy or (acceptance and commitment therapy)).ti.
2 (web or online or internet or virtual or app or cyber).ti.
3 (medical students or residents or residency students).ti.
4 1 and 2 and 3

## 9. PUBMED

((((mindfulness[Title]) OR (cognitive behavioral therapy[Title])) OR (acceptance[Title] AND commitment therapy[Title]) AND (2010:2020[pdat])) AND ((((((web[Title]) OR (virtual[Title])) OR (cyber[Title])) OR (internet[Title])) OR (app[Title])) OR (online[Title]) AND (2010:2020[pdat]))) AND (((medical students[Title]) OR (residents[Title])) OR (residency students[Title]) AND (2010:2020[pdat]))

# Appendix 2: Review Protocol

## 1) Search question or objective

The question is the following: “How effective are online mindfulness interventions to address medical students’ mental health?”

## 2) Inclusion/exclusion criteria

Inclusion criteria

- Types of studies: all types of studies
- Participants: medical students or residents
- Interventions: online mindfulness

Exclusion criteria

- Interventions not delivered online
- participants not medical students
- Language not English
- Not full text available
- Excerpts from books

## 3) Timeframe

- Last 10 years (given that research on online mindfulness started in year 2013 based on a previous systematic review[10])

## 4) Databases to be searched

- ProQuest, Medline, PubMed, PsycINFO, Web of Science, IEEE Explore, Cochrane, and CINAHL

## 5) Search strategy

The following search strategies should be followed.

## ProQuest

S4 ((ti(Medical students) OR ti(residents) OR ti(residency students)) AND PEER(yes)) AND ((ti(cyber) OR ti(web) OR ti(virtual) OR ti(online) OR ti(app) OR ti(internet)) AND PEER(yes)) AND ((ti(mindfulness) OR ti(cognitive behavioral therapy) OR ti(acceptance AND commitment therapy)) AND PEER(yes))

S3 ti(mindfulness) OR ti(cognitive behavioral therapy) OR ti(acceptance AND commitment therapy)Limits applied

S2 ti(cyber) OR ti(web) OR ti(virtual) OR ti(online) OR ti(app) OR ti(internet)Limits applied

S1 ti(Medical students) OR ti(residents) OR ti(residency students)Limits applied

## Medline

1 Students, Medical/ or Education, Medical, Undergraduate/ or Education, Medical, Graduate
2 limit 1 to (english language and yr="2010 - 2020")

3 Cyber/ or Internet/ or Web Browser/ or virtual/ or online/ or app/ or internet/

4 (acceptance and commitment therapy).mp. [mp=title, abstract, original title, name of substance word, subject heading word, floating sub-heading word, keyword heading word, organism supplementary concept word, protocol supplementary concept word, rare disease supplementary concept word, unique identifier, synonyms]
5 1 and 2 and 3 and 4

## PsychINFO

((ti(Medical students) OR ti( residents) OR ti(residency students )) AND PEER(yes)) AND ((ti(cyber) OR ti(web) OR ti(virtual) OR ti(online) OR ti(app) OR ti(internet)) AND PEER(yes)) AND ((ti(mindfulness) OR ti(cognitive behavioral therapy) OR ti(acceptance AND commitment therapy)) AND PEER(yes))

## Web of Science

# 4 #3AND #2 AND #1
# 3 (TI=(mindfulness OR cognitive behavioral therapy OR acceptance "and" commitment therapy) ) AND LANGUAGE: (English)

# 2 (TI=(cyber OR web OR virtual OR online OR app OR internet) ) AND LANGUAGE: (English)

# 1 (TI=(Medical students OR resident* OR residency student*) ) AND LANGUAGE: (English)

## CINAHL

S4 S1 AND S2 AND S3
S3 TI medical students OR TI residents OR TI residency students
S2 TI cyber OR TI web OR TI virtual OR TI online OR TI app OR TI internet
S1 TI mindfulness OR TI cognitive behavioral therapy OR TI (acceptance and commitment therapy)

## IEEE Xplore

4 [((((((Mesh_Terms:mindfulness) OR Mesh_Terms:cognitive behavioral therapy) OR Mesh_Terms:acceptance and commitment therapy) refined by:Year:2010-2020 )) AND (((((((Mesh_Terms:web) OR Mesh_Terms:cyber) OR Mesh_Terms:online) OR Mesh_Terms:web) OR Mesh_Terms:app) OR Mesh_Terms:internet) refined by:Year:2010-2020 )) AND ((((Mesh_Terms:medical students) OR Mesh_Terms:residents) OR Mesh_Terms:residency students) refined by:Year:2010-2020 )](https://ieeexplore-ieee-org.ezproxy.library.yorku.ca/search/searchresult.jsp?contentType=all&sortType=&searchField=Search_All&combineQuery=ranges%3D2010_2020_PublicationYear%26matchBoolean%3Dtrue%26searchField%3DSearch_All%26queryText%3D%28%28%28meshTerms%3Amindfulness%29+OR+meshTerms%3Acognitive+behavioral+therapy%29+OR+meshTerms%3Aacceptance+and+commitment+therapy%29.OPAND.ranges%3D2010_2020_PublicationYear%26matchBoolean%3Dtrue%26searchField%3DSearch_All%26queryText%3D%28%28%28%28%28%28meshTerms%3Aweb%29+OR+meshTerms%3Acyber%29+OR+meshTerms%3Aonline%29+OR+meshTerms%3Aweb%29+OR+meshTerms%3Aapp%29+OR+meshTerms%3Ainternet%29.OPAND.ranges%3D2010_2020_PublicationYear%26matchBoolean%3Dtrue%26searchField%3DSearch_All%26queryText%3D%28%28%28meshTerms%3Amedical+students%29+OR+meshTerms%3Aresidents%29+OR+meshTerms%3Aresidency+students%29&history=no) You Refined By: Year: 2010-2020

3 [((("Mesh_Terms":medical students) OR "Mesh_Terms":residents) OR "Mesh_Terms":residency students)](https://ieeexplore-ieee-org.ezproxy.library.yorku.ca/search/searchresult.jsp?contentType=all&ranges=2010_2020_PublicationYear&matchBoolean=true&searchField=Search_All&queryText=(((meshTerms:medical+students)+OR+meshTerms:residents)+OR+meshTerms:residency+students)&history=no) You Refined By: Year: 2010-2020

2 [(((((("Mesh_Terms":web) OR "Mesh_Terms":cyber) OR "Mesh_Terms":online) OR "Mesh_Terms":web) OR "Mesh_Terms":app) OR "Mesh_Terms":internet)](https://ieeexplore-ieee-org.ezproxy.library.yorku.ca/search/searchresult.jsp?contentType=all&ranges=2010_2020_PublicationYear&matchBoolean=true&searchField=Search_All&queryText=((((((meshTerms:web)+OR+meshTerms:cyber)+OR+meshTerms:online)+OR+meshTerms:web)+OR+meshTerms:app)+OR+meshTerms:internet)&history=no) You Refined By: Year: 2010-2020

1 [((("Mesh_Terms":mindfulness) OR "Mesh_Terms":cognitive behavioral therapy) OR "Mesh_Terms":acceptance and commitment therapy)](https://ieeexplore-ieee-org.ezproxy.library.yorku.ca/search/searchresult.jsp?contentType=all&ranges=2010_2020_PublicationYear&matchBoolean=true&searchField=Search_All&queryText=(((meshTerms:mindfulness)+OR+meshTerms:cognitive+behavioral+therapy)+OR+meshTerms:acceptance+and+commitment+therapy)&history=no) You Refined By: Year: 2010-2020

## COCHRANE

1 (mindfulness or cognitive behavioral therapy or (acceptance and commitment therapy)).ti.
2 (web or online or internet or virtual or app or cyber).ti.
3 (medical students or residents or residency students).ti.
4 1 and 2 and 3

## PUBMED

((((mindfulness[Title]) OR (cognitive behavioral therapy[Title])) OR (acceptance[Title] AND commitment therapy[Title]) AND (2010:2020[pdat])) AND ((((((web[Title]) OR (virtual[Title])) OR (cyber[Title])) OR (internet[Title])) OR (app[Title])) OR (online[Title]) AND (2010:2020[pdat]))) AND (((medical students[Title]) OR (residents[Title])) OR (residency students[Title]) AND (2010:2020[pdat]))

## 6) Methodology for data extraction and analysis

- The two authors will independently screen titles and abstracts of the search results to decide whether a reference meets the inclusion criteria or should be excluded. Disagreements between authors will be resolved through discussion.
- After first screening of the titles and abstracts and resolving disagreements full–text articles will be retrieved to make a final decision on inclusion/exclusion of each article.
- For each included article, relevant data will be extracted using a structured excel form developed by the team. The excel will be used to produce PICO table and compare the included studies. It will also facilitate ethe assessment of the risk of bias
- The excel data will be collected first by VY will be reviewed and then verified for completeness and accuracy by CE.
- The following types of bias will be screened for confounding, selection bias, exposure assessment, outcome measurement, and missing data.
